# A natural language interface plug-in for cooperative query answering in biological databases

**DOI:** 10.1186/1471-2164-13-S3-S4

**Published:** 2012-06-11

**Authors:** Hasan M Jamil

**Affiliations:** 1 Department of Computer Science, Wayne State University, Michigan, USA

## Abstract

**Background:**

One of the many unique features of biological databases is that the mere existence of a ground data item is not always a precondition for a query response. It may be argued that from a biologist's standpoint, queries are not always best posed using a structured language. By this we mean that approximate and flexible responses to natural language like queries are well suited for this domain. This is partly due to biologists' tendency to seek simpler interfaces and partly due to the fact that questions in biology involve high level concepts that are open to interpretations computed using sophisticated tools. In such highly interpretive environments, rigidly structured databases do not always perform well. In this paper, our goal is to propose a semantic correspondence plug-in to aid natural language query processing over arbitrary biological database schema with an aim to providing cooperative responses to queries tailored to users' interpretations.

**Results:**

Natural language interfaces for databases are generally effective when they are tuned to the underlying database schema and its semantics. Therefore, changes in database schema become impossible to support, or a substantial reorganization cost must be absorbed to reflect any change. We leverage developments in natural language parsing, rule languages and ontologies, and data integration technologies to assemble a prototype query processor that is able to transform a natural language query into a semantically equivalent structured query over the database. We allow knowledge rules and their frequent modifications as part of the underlying database schema. The approach we adopt in our plug-in overcomes some of the serious limitations of many contemporary natural language interfaces, including support for schema modifications and independence from underlying database schema.

**Conclusions:**

The plug-in introduced in this paper is generic and facilitates connecting user selected natural language interfaces to arbitrary databases using a semantic description of the intended application. We demonstrate the feasibility of our approach with a practical example.

## Background

The choice of interface becomes a critical factor in many large community databases such as GenBank, UCSC Genome Browser, and FlyBase, as well as many smaller individual databases, used by researchers across the globe. Applications in life sciences domain are often designed using specific analysis or query need in mind. These applications widely use graphical interface technologies for access to the underlying database and to capture query semantics. These query driven graphical user interfaces do not allow free form and arbitrary querying, thereby limiting the use of the underlying database content. Since these interfaces must necessarily be built for each query need, cost acts as a significant prohibitive factor. As a result of adopting pre-fabricated web interfaces for access, the wealth of these repositories remain poorly used. To compensate for the perceived lack of use and to serve users who desire to exploit the content in ways other than originally anticipated, database contents are often made available for copying and differential use. This materialized view [1] based alternative has been proven to be expensive and complicated, principally because indiscriminate copying introduces version maintenance problem, making it difficult to correct obsolete information in the copied version when the main copy is corrected. End users in general, and biologists in particular, prefer to use graphical interfaces to access databases mainly because query language based interfaces demand considerable familiarity with the underlying database schema and expertise in framing semantically meaningful queries. While SQL or XQuery type query languages allow ad hoc and arbitrary querying, and potentially eliminate the bottleneck imposed by pre-fabricated graphical interfaces, biologists are resistant to adopting such an archaic interface. To exacerbate this situation, databases are slowly but certainly adopting more complex XML type representations for the purpose of information interchange and data integration, making it even harder for biologists to accept textual query interfaces because XQuery type languages are considerably more complex than SQL.

Regardless of the query platform used, in works such as BioBike [2], it was argued that for several decades biologists have voted overwhelmingly not to embrace computer programming as a basic tool through which to look at their world. BioBike tries to simulate a natural language like interface using graphical interactions as a means to support access to database content. The idea is based on the premise that the limitations imposed by graphical interfaces and the complexity inherent in query languages such as SQL and XQuery can be bypassed if access to information is facilitated using natural language queries. Natural language processing (NLP) based query answering systems enjoy the benefit of user's natural ability to frame queries at a conceptual level she is comfortable with. The real issue is how abstract such queries can be so that the user need not worry about the technicality involved in framing the query for the database query engine to understand and execute the intended query. The higher the abstraction level, the harder usually it is to parse and map the query on the underlying database schema. Such a high level interface still remains illusive mostly because of the representation hurdles, highly interpretive nature of life sciences data (tool applications are often essential before an understanding can be gained for many health care data. Simple read off of the data as in relational model does not reveal any information in general), translation from natural language to database queries, and the inherent difficulty in processing NLP queries. A practical approach, we argue, is providing a flat universal relational view of data so that users can comprehend and query the information repositories at their disposal. Once chosen, the question remains, how do we facilitate comprehension of a natural language query in a given context, device a strategy to compute its response, and implement the strategy as a traditional query over structured relations stored in local or remote information repositories.

Our goal in this paper is to develop a semantic "plug-in" in the form of a common knowledge ontology to describe the intended semantics of the underlying database, still at a conceptual level. We use this common knowledge ontology to bridge the semantic gap between the database schema and the NLP parsing engine so that meaningful connections between the natural language queries and database can be established. We present a method to map conceptual queries to database schema and show that a natural language parser now has a better chance to generate meaningful SQL queries, especially for short natural language queries. We also show that the use of the plug-in makes it possible for the parsing systems to be unaware of low level schema details.

### Related research

While systems such as Toorjah [3] and CoIn [4] attempt to improve the usability repertoire of databases, they still do not support unrestricted use as they remain committed to pre-fabricated query interfaces. In this paper, we argue that universal and ad hoc access to community biological databases can be facilitated through natural language interfaces to counter interface constraints. We envision database independent natural language interfaces which will intelligently map English sentences to SQL like structured queries to return a response. In such an environment, we also expect the system to tolerate discrepancies in users' knowledge of the database scheme and to allow flexibility by not demanding strict schema adherence in user queries.

In natural language processing community, Stanford Parser [5-7] and MINIPAR [8] are two leading tools that perform fairly well in recognizing acceptable English sentences. The success of NLP systems such as these are encouraging database interface development in English to facilitate access to databases using text querying [9,10] with limited scope. While NLP interfaces for traditional databases has been extensively studied in the literature (e.g., [11-17]), several recent proposals toward mapping NL queries to SQL [18-21] are of significant interest. However, most of these interfaces work well for a limited class of queries, and for a specific database schema. In other words, changes in application objectives or underlying databases are extremely difficult to accommodate in these approaches. An important limitation is that these systems are unable to incorporate arbitrary rules that drive computational tools to make inferences, a feature that biologists need. Our contention is that although there have been many attempts in other domains, natural language interfaces for biological databases remain an emerging area of research. The few that are prominent (e.g., BioBike [2]), are extremely rigid and difficult to replicate.

### An illustrative example

**Figure 1:**
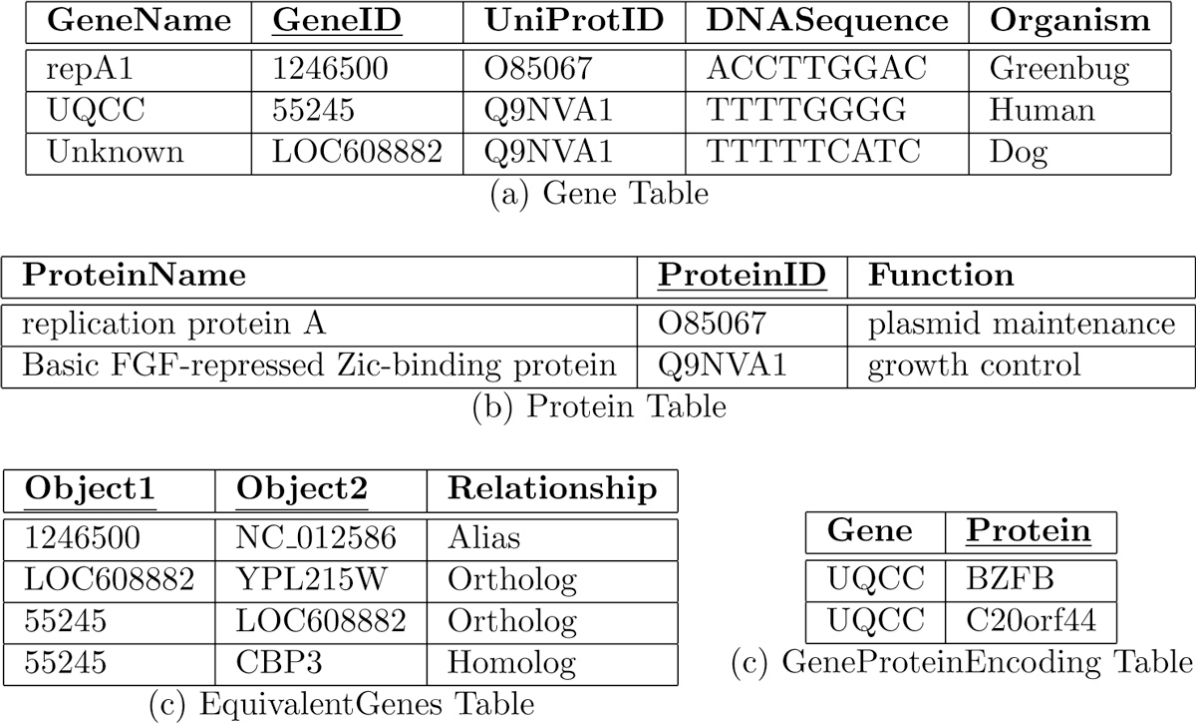
**Example database Δ**.

Consider the biological database shown in Figure 1 consisting of relationships among genes and organisms, and a set of computational tools to analyze its contents. The database has the following tables with the primary keys underlined - *Gene *(represents genes and their properties such as common name, ID, UniProt [22] ID, underlying DNA sequence and the organism in which it is found), *Protein *(represents proteins and their properties such as common name, ID and functions), *EquivalentGenes *(represents an equivalence relation between two biological concepts and their relationships), and *GeneProteinEncoding *capturing all proteins encoded by a gene.

The database may also include computational tools such as DAVID [23] and GeneCards [24] for ID mapping, and BLAST [25] for sequence homology analysis. These tools are used to analyze the contents in more sophisticated ways using generalized knowledge such as the ones below.

1. Function of gene *A *is equivalent to function of gene *B *IF *A *and *B *are equivalent.

2. Two object IDs are equivalent IF they are aliases, orthologs, or homologs.

3. Two sequences are homologs IF BLAST returns true (given a threshold *θ*).

4. Two IDs are homologs IF their corresponding sequences are so.

5. Two IDs are equivalent IF DAVID or GeneCards returns one for the other.

6. Two IDs *A *and *B *are equivalent IF a gene (ID *A*) encodes a protein (ID *B*).

These rules can be used to derive the function of gene *LOC458201 *in *chimpanzees *(using rule 1) even though there is no record of this gene in the database. This is because *LOC458201 *is an ortholog of the gene *UQCC *in *humans*, and either GeneCards will successfully establish the correspondence (using rule 5), or Blast will (using rule 3). Users traditionally write queries and apply similar functions to draw conclusions. The question that we are trying to address is: is it possible for a natural language interface to hide the complex computational view of the database and respond to queries from a more conceptual level For example, could we develop a system to answer natural language query such as (1) *"what is the function of gene LOC458201 " *or (2) *"which gene in chimpanzees is responsible for growth control " *Our focus in this paper is to propose a practical method to map such queries to semantically equivalent database specific SQL queries.

We also envision that a generalization of the above mechanism will significantly enhance the query answering capability of a database when augmented with appropriate deductive capabilities and machineries to compose scripts for executing computational pipelines. For example, the query (2) above cannot be directly computed from the example database in Figure 1. To compute this query, we must first find out the complete set of *chimpanzee *genes and apply the six rules above to infer possible responses. If, in addition to the above six rules, we also know that the HomoloGene database [26] can be queried to get a list of organism genes, we can then construct a pipeline to compute a possible response and find that *chimpanzee *gene *LOC458201 *is a growth control gene. Alternately, we can find the growth control genes of all organisms, and find out from GeneCards if there is a *chimpanzee *ortholog for any of these genes. In the remainder of this paper, we discuss a general approach toward constructing a computational strategy to respond to a natural language query. Our focus is mainly on strategy construction and query translation by exploiting the strengths of MINIPAR [8], or other similar tools for parsing, and dependency assignments of query sentences. Accordingly, we discuss the following components needed for the construction of our interpreter.

1. A model for an ontology with deduction rules is proposed. Such an ontology helps bridge the semantic gap between the decoupled user view and the database view. Earlier research have already exploited similar adoption to facilitate natural language interfacing [27].

2. The concept of a schema graph to merge the ontology and the database scheme as an interface between the user view of the database and the database instance. Similar tools, e.g., structured object model [28], for bridging user view and the database have been previously investigated and exploited.

3. We adopt a deductive query processing model to support intensional query processing. But we do so using SQL since the vast majority of biological databases use a relational platform. We, however, derive the computational strategy intensionally entailed by the query using deductive rules in the ontology. A similar approach, though in significantly limited form, has been exploited in BioBike [2].

4. We support querying online resources to access tools and databases needed to process user queries and modeled as functions in the ontology. We exploit the notions of remote user defined functions [29] and biological workflows [30] we proposed earlier to facilitate such integration in a user transparent way.

In the next few sections, our plan is to explain these components using the query *"List all growth control genes in dogs that function similar to human genes (but are absent in fruit flies)." *For simplicity, we first consider the query without the parenthesized part, and then consider the full query to see how more complex queries can be constructed. We discuss the construction of structured queries in the context of the database in Figure 1.

## Methods

### Structure of a natural language interpretable database

The overall approach is to view the base database as a collection of entity and relationship sets in the sense of ER model [31], and preserve this conceptual view across different levels of abstractions we use to facilitate mapping of natural language queries to SQL queries. Accordingly, the base database Δ is a set of 3rd normal form classical relations *r *over schemes *R *with attributes *A*_1_, . . ., *A_n _*with corresponding domains *D*_1_, . . ., *D_n_*. The database Δ in Figure 1 contains four such relations wherein the primary key of each relation is underlined. Although not readily apparent, the relations *EquivalentGenes *and *GeneProteinEncoding *are relationship sets in the sense of ER model while the remaining two are entity sets. For this fact to be available to the natural language interpreter and to exploit this structural information toward query construction, we complement the database with the following machineries.

### Common knowledge ontology

**Figure 2:**
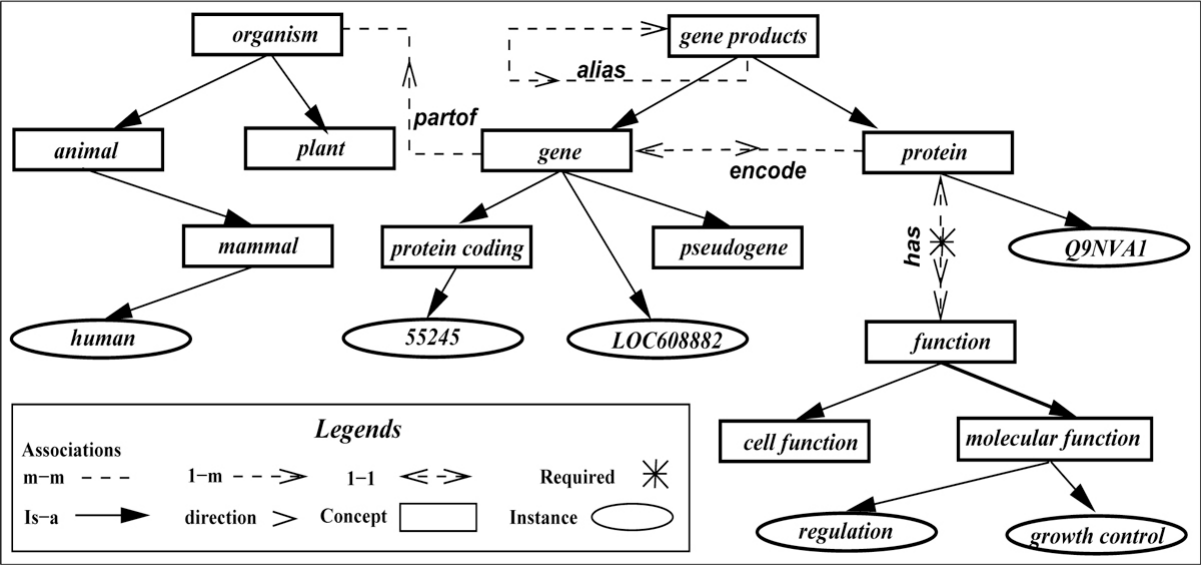
**Ontology  O for database Δ in Figure **1.

An ontology O  in our system is a set of basic concepts  C, a set of base relationships  R among the concepts, a set of derived relationships as inference rules  I involving ontological concepts in  C, and a type hierarchy  T involving the concepts in  C. Inference rules thus define more concepts in the form of relationships involving the concepts. In Figure 2, we show such an ontology for our database Δ. In this figure, *genes, functions *and *organisms *are concepts, and so are *molecular functions *and *plant. partof *and *has *are relationships. In this system, concepts are distinct from instances or objects, and instances are not part of the ontology although we show a few instances (as ellipses) for expository purposes, e.g., *human *is an instance of a concept. For every concept and relationship in the ontology, we expect to find a representation of each as an entity or relationship set, or as their attribute in the underlying database. The converse need not be true, i.e., we may have attributes and relations in the database that are not represented in the ontology.

The knowledge similar to the six rules embedded in the database Δ can be represented in terms of the concepts in the ontology  O as the following set of rules. In this ontology language, all predicates are either unary, or binary. The object (or instance) of a concept is represented in the form of *concept(***x***) *where *concept *is the concept name, and **x **is either a variable or a concrete object (constant) that uniquely identifies an object in the database. Similarly, a relationship between two concepts is represented as a binary predicate of the form *relationship(***x**, **y***) *as all relationships in our ontology are binary in nature.

1. *function(X, Y) ← alias(X, Z), has(Z, Y)*.

2. *alias(X, Y) ← homolog(X, Y)*.

*alias(X, Y) ← ortholog(X, Y)*.

*alias(X, Y) ← gene(X), encode(X, Y)*.

Note that the concepts *homolog *and *ortholog *in the rules above are not represented in Figure 2, yet they are part of this vocabulary. Concepts not part of the ontology description are expected to be computed or represented in the database as properties of entities or relationships. All unary undefined concepts are considered entity properties while binary concepts are considered relationship properties. Finally, observe that not all six rules are captured in the ontology as some of them are more database and operation specific. Our goal is to cover this gap using a reduction function, defined next.

### Ontology augmented schema graph

**Figure 3:**
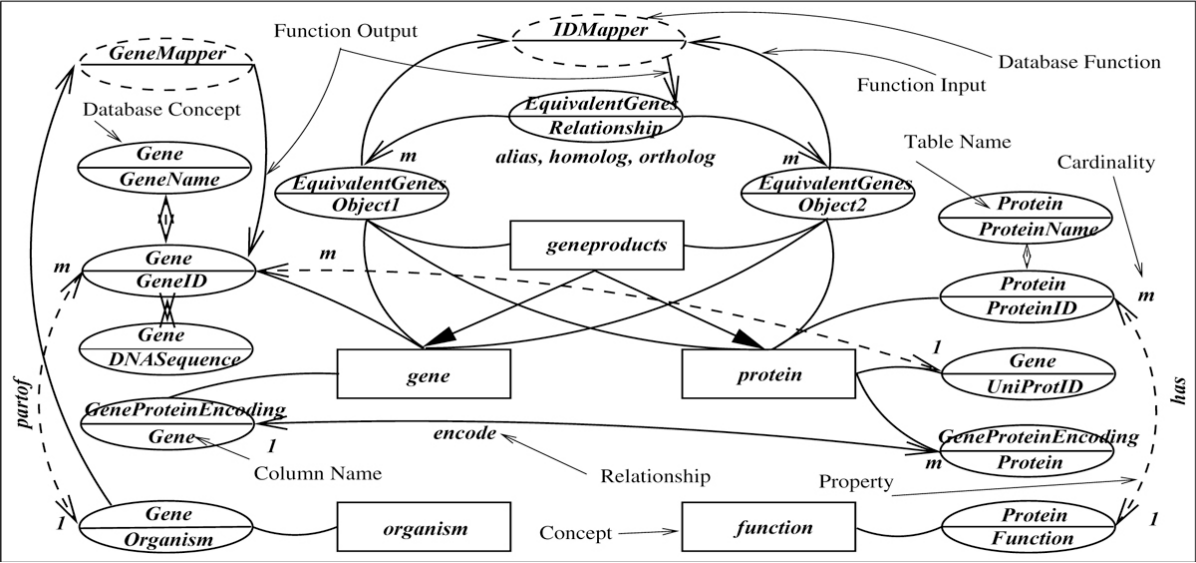
**Schema graph of database Δ, ontology  O and type system  T**.

The ontological characterization of a database is at the highest level of conceptual relationship among the database objects. It is defined independent of the database schema and structure so that it reflects a commonsense and general view of the information content. This view is expected to be close to the natural language queries we anticipate. To bridge this high level view with the underlying database, we create a *schema graph *that merges the ontology with the database scheme to capture the semantic relationships the database objects share through their attributes to facilitate natural language query to SQL translation. Before presenting the process of conversion from ontology to schema graph, we present the idea on intuitive grounds. Given the database scheme ∑ = ⋃_*r*∈Δ_*r*(*R*), the schema graph for the database Δ in Figure 1 with respect to the ontology  O in Figure 2 is shown in Figure 3.

In the schema graph, (i) a rectangle represents the concept in the ontology, (ii) a solid ellipse represents a database attribute in which the annotation above the middle line is the relation name and the one below is the attribute name, (iii) a solid line between an ellipse and rectangle represents the attribute's classification in  T, (iv) a solid named double ended arrow between two ellipses represents an association in  O, and (v) a dashed unnamed double ended arrow represents a property of an object. A value concept is represented in two ways - (vi) as an extension in a database column (shown as a solid ellipse relating one or two database attributes, e.g., *relationship *in *EquivalentGenes*), or (vii) as an intension modeled as a function that returns a value in a database column (shown as a dashed ellipse accepting inputs of database attribute types, e.g., the unary function *GeneMapper *which returns *GeneID *type values, and the binary function *IDMapper *returning *Relationship *type values).

The schema graph emphasizes the concepts, and groups database attributes according to them. The relationships (associations, property, and value mappings) are captured using attribute relationships. Additional database attributes not present in the ontology are also included as properties of a concept represented as keys. Finally, user defined database functions are included to complete artifacts needed for querying the database. Essentially, a schema graph represents reachability structure among the database concepts, using which we plan to construct SQL queries from natural language sentences.

### Procedure for generating the schema graph

In the spirit of [32,33], we make no distinction between objects represented as values, attributes or classes, and adopt the view that ontological concepts not only relate class objects, but also value objects. For example, *gene(55245) *represents the object *55245 *in the table *Gene *in Figure 1 as the class object *gene *in ontology  O. However, the fact that *UQCC *is an *ortholog *of *LOC608882 *is not captured as a relationship in table *EquivalentGenes*. It is however captured as an attribute value of the relationship. To accommodate such relationships, we adopt a priority scheme for mapping concepts to underlying database in which we prefer to map them first to the database scheme, and then we map only those concepts to instances for which we fail to find a schema level mapping. We explain the process below the result of which is the schema graph shown in Figure 3 on database Δ and ontology  O.

#### Mapping ontology to schema graph

We define two functions *μ *and *φ *to map respectively the concepts and associations in the ontology to an underlying database. Let ℜ and  A correspondingly be finite sets of relation and attribute names in database Δ where each attribute name in  A is made unique by prefixing the relation name to which it belongs, i.e., for relation *r *∈ ℜ and *A *∈ *r*(*R*), there is r.A∈A. Then, $\mu :C\to 2^{A}$, such that for $\forall c\forall c^{\prime }\forall s\forall t\left(c,c^{\prime }\in C\wedge s\in \mu \left(c\right)\wedge t\in \mu \left(c\prime \right)\wedge s=t\Rightarrow c=c\prime \right)$. In other words, all concept to attribute mappings are unique. For example, *μ*(*gene*) = {*Gene.GeneID, EquivalentGene.Object1, GeneProteinEncoding.Gene*}, and that *Gene.GeneID *∈ *μ*(*gene*) and *Gene.GeneID *∈ *μ*(*protein*) is not simultaneously possible. Also, whenever ∃*s, s *= *r.A *∈ *μ*(*c*), and *A → R *holds, concept *c *is said to have an *entity mapping c ⇀ r*. The set of all concepts having entity mapping is denoted by  E, and nothing else is in  E. In the gene database, *μ*(*gene*) has an entity mapping since *GeneID *in *Gene *is a primary key. The mappings that are not in  E, i.e., mappings ∀c∈O, *μ*(*c*) *≠ *∅ but c∉E, are called *property mapping*. Generally, all mappings in *μ*(*c*) are denoted as *c *↠ *r.A*.

For every association a∈R involving concepts $c,c^{\prime }\in C$, if (i) $\left\{c,c^{\prime }\right\}\subseteq E$ (this means *c *and *c' *are primary keys of relations other than *r*, because they cannot be simultaneously keys for *r*) and *r.A *∈ *μ*(*c*) ^ *r.B *∈ *μ*(*c'*) and *r.A *and *r.B *are prime attributes (part of a primary key) of *r*, or (ii) *c *= *r*. A∈E, *c*' = *r*'. B∉E and *r *= *r*', then *a *has a *relationship mapping a *⇁ 〈*r.A, r.B*〉. The set of all associations having relationship mapping is denoted by  S. For example, in the first category, for the associations *alias *and *encode *respectively, *alias *⇁ 〈*EquivalentGenes.Object1, EquivalentGenes.Object2*〉 and *encode *⇁ 〈*GeneProteinEncoding.Gene, GeneProteinEncoding.Protein*〉 hold. In the second category, for the associations *partof *and *function partof *⇁ 〈*Gene.GeneID, Gene.Organism*〉 *function *⇁ 〈*Protein.ProteinID, Protein.Function*〉 hold.

To facilitate mapping of concepts that are captured as values at the database level, we use a second function *φ*. Therefore for every concept c∈C such that *μ*(*c*) = ∅, *φ *maps *c *to a set of instance values in relations, i.e., $\varphi :C\to 2^{V}$, where  V is a set of unique values of the form *r.A.v *in which *r *is the relation name, and *v *is a value in column *A *of *r *(in the parlance of [34], the representation corresponds to *r*[*A → v*]), i.e., *→ σ*_*A *= *c*_(π_*A*_(*r*)) *≠ *∅. Since value concept may be part of an entity set as well as a relationship set, it is denoted as a polymorphic relation *c *↣ 〈*r.A, r.B*〉 whenever ∃*a, a *⇀ 〈*r.A*〉 and *r.B.v *∈ *φ*(*c*), or *c *↣ 〈*r.A, r.B, r.C*〉 whenever ∃*a, a *⇁ 〈*r.A, r.B *〉 and *r.C.v *∈ *φ*(*c*).

The predicate representation of value concepts is identical to class concepts, i.e., *gene(X), function(X) *or *ortholog(X, Y)*. In this database, the class concept *gene *is represented as a table (Figure 1(a)), whereas the class concept *function *is represented as an attribute of the table *Protein*. In both representations, *X *is the primary key of the tables *Gene *and *Protein*, respectively. For example, for *gene(55245), 55245 *is a primary key, and for *ortholog(UQCC, LOC608882)*, both *UQCC *and *LOC608882 *are in the primary key of *EquivalentGenes *table. The set of all such mappings are denoted by  P.

##### Concept and association mapping functions

Implementation of the concept mapping function *μ*, and value mapping function *φ *is quite straight forward and intuitive. For the function *μ*, first, we use a subset of the Gene Ontology [35] and adopt the ontology as our type hierarchy  T. We use a simple syntactic scheme for typing attribute values. For example, *UQCC *will be typed as a *gene name*, while *55245 *will be recognized as a *gene*. We inspect and select a representative set of values per column of each table in the database, using a statistical sampling technique. The type of the attribute is then taken as the least upper bound of all the types of sampled values in each column in the type hierarchy  T. The value concept mapping function *φ *is implemented as *σ*_*A *= *c*_(π_*A*_(*r*)) for each attribute *A *of each relation *r *in Δ. With the help of these two functions, we classify each attribute of each relation as a type/class concept in  T, and capture the value concepts in  O, i.e., concepts that do not have an attribute level correspondence, as associations. For example, *ortholog *can be viewed as a relationship between two gene products *X *and *Y *, whereas *growth control *may be viewed as a unary relationship with a protein *X*, a property.

#### Database to schema graph mapping

Since users view of the database is somewhat unrestricted and independent of any database schema, or the assumed ontology, queries may involve concepts and relationships that are represented in the database even though they are not present in the ontology. Therefore, the ontology to schema graph mapping described in the previous section will miss them. To fill this gap, we also define a backward mapping from the database to schema graph as follows.

Let *r*(*R*) be a relation with primary key *K *such that *K *is mapped to concept *c*, i.e., *r.K *∈ *μ*(*c*). If c∈O, *r.K *will be connected to a concept *c *in schema graph as described with a solid line although it may not have any relationship with any other concept. However, if *r.K *∈ *μ*(*c*) but c∉O, we create a new concept *c *in the schema graph and represent it. For both cases, i.e., c∈O and c∉O, every attribute *A *∈ {*R *- *K*} that is not in  O but *r.A *∈ *μ*(*c*) for some *c*, we create a concept *c *and associate *r.A *with *c*, and connect *r.A *as an unnamed property of *r.K *(dashed unnamed double arrow). In the schema graph in Figure 3, *GeneName *and *ProteinName *are of concept type *name *(not shown), and *DNASequence *is of concept type *sequence *(also not shown). These are called database concepts and always connect to the concept, representing the key of the relation they belong to.

For every database function *f*, we add an intensional value concept (dashed ellipse) that accepts a set of concepts and returns a concept in the schema graph. For example, *GeneMapper *and *IDMapper *are two such functions. Since these database functions produce value concepts, value concepts in *O *can be mapped to these functions as well. For example, *IDMapper *is an implementation of the value concepts in column *Relationship*, i.e., given two gene products, it returns the nature of their relationship.

### Query transformation

While natural language interfaces are powerful, intuitive and desired, it is well established that current technology does not make it possible for us to support unrestricted querying on all databases accurately [36,37]. It is thus often suggested [38,39] that limiting the type of queries supported on a specific database may help alleviate the problem and improve the usability of such interfaces over generic databases. Accordingly, our goal in this paper is to allow three types of queries - interrogative, imperative and declarative. While all three types of sentences are basically queries, an interrogative sentence poses a question specifically using a structure such as "Does", "Is", "Why", "How", "What", and so on. While "Does" and "Is" types of questions mainly pose existential or verification type queries and can be computed using selection operation in relational algebra, "Why" and "How" types of questions often imply deductions and require significant reasoning to answer. Imperative questions, and often declarative questions, generally use structures such as "List", "Print", and "Return" that is a selection query in its simplest form. The real complexity though lies in the way such questions are formed.

### Semantic roles of objects in queries

**Figure 4:**
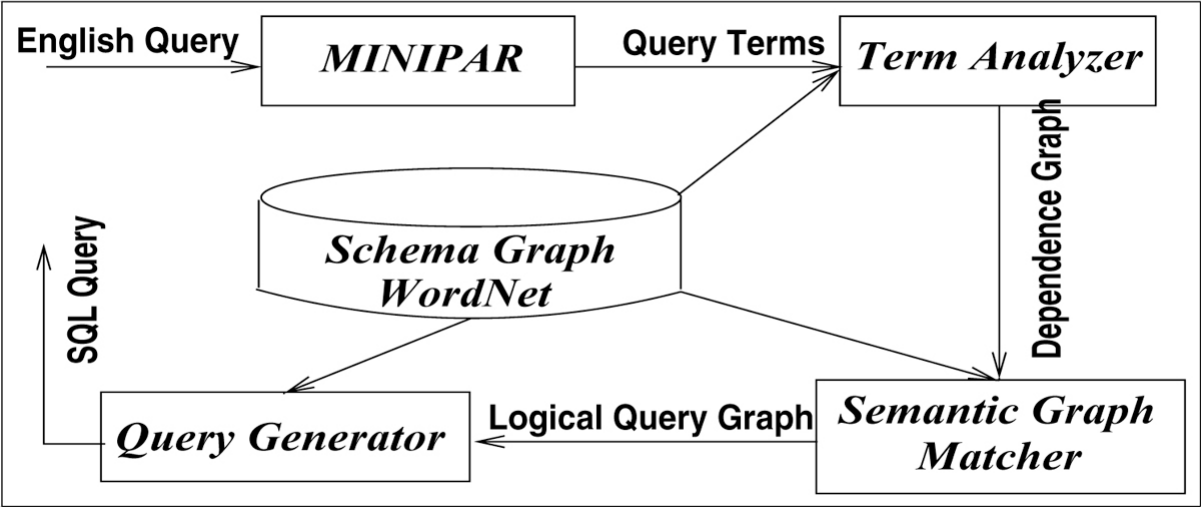
**Query processing and transformation model**.

Since our main interest is in mapping natural language queries to structured queries, and not in developing parsing technologies, we exploit systems such as MINIPAR [8] or Stanford Dependence Parser [40] that are widely respected for their accuracy in parsing query sentences and computing dependence graphs and semantic roles. We accept the semantic roles generated by MINIPAR in conjunction with data dictionaries such as WordNet [41]. The overall translation process is shown in Figure 4 in which we introduce three components - term analyzer, semantic graph matcher, and query generator.

The term analyzer generates the semantic role of the query sentence in the form of a dependence graph as closely as possible, using the concepts and terms in the schema graph, synonyms and other equivalence relations in WordNet and the type hierarchy  T. Intuitively, the term analyzer transforms the query sentence using terms in the schema graph concepts. The dependence graph is analyzed to identify the subgraphs in the schema graph that match. If the match results in a connected graph, it is accepted as a logical query graph and sent to query generator. The query generator then generates the SQL query by properly sequencing the joins, plugging selection conditions, and substituting function calls when needed. Let us revisit the query we introduced in section, *"[List all growth control genes in dogs] that function similar to human genes (but are absent in fruit flies)." *and discuss it in the context of the machineries we have introduced so far, and see how we can formulate a structured query to respond to it, and how the nature of the query changes as we modify it slightly. In this query, we have separated four segments (underlined, within square brackets, up to the parenthesis, and the whole sentence) that changes the nature of the query when added successively. The query *"List all growth control genes" *is a simple imperative query that can be answered with a simple selection query as follows:

select *GeneID*

from *Gene G, Protein P*

where *G.UniProtID = P.ProteinID *and *P.Function = "growth control"*

**Figure 5:**
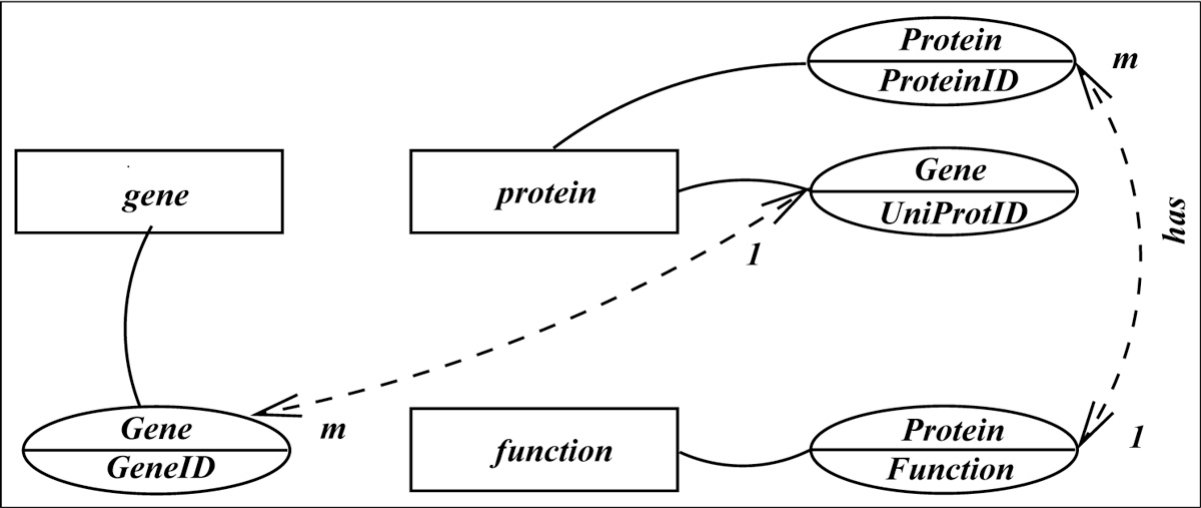
**Logical query graph**.

This query can be generated based on dependence parse tree of the query sentence and by establishing the semantic roles of the sentence. For example, MINIPAR will generate the terms {*(list.v)(growth.n)(control.n)(genes.n)*} where *"growth control" *is treated as a modifier of the head noun *gene*. We then match the head noun with the concept *gene*, and *"growth control" *to the concept *function *using type hierarchy  T. The relationship between these two terms are then generated as the graph in Figure 5 from the schema graph, called the *logical graph *(a subgraph of the schema graph), since this is the shortest path to connect them (*gene → Gene.GeneID → Gene.UniProtID → protein → Protein.ProteinID → Function*). Semantically this subgraph means every gene has a UniProt protein ID for which a function is available. In terms of the database Δ, this graph also entails the SQL query above. While this query is somewhat straightforward, the query *"List all growth control genes in dogs" *is not simple to generate, and will require substantial machineries described next. The query *"List all growth control genes in dogs that function similar to human genes" *will require even more.

### Correspondence between English sentence structures and concepts

Once the sentence is parsed and terms are generated by MINIPAR, the query is decomposed into semantic roles and verbs. Each semantic role is comprised of a head noun and a set of modifiers. In the query, *"List all growth control genes", growth control *(both nouns) are modifiers of the head noun *gene*. The verbs are the terms that relate the semantic roles we generate. Once the semantic roles for the English sentence has been established, and the dependence graph has been generated by the term analyzer, we use table 1 as a guide toward establishing correspondence between the terms and the concepts in the schema graph.

**Table 1:** Correspondence table

**POS**	**Ontological entity**
Common noun	Concept, property of a concept
Proper noun	Object or head noun
Transitive verb	Association name
Adjective	Property of concept or object
Adverb	Property of association

### A generalized mapping procedure

The SQL query that our system would ordinarily generate (in the absence of the rules in the ontology) to compute the query *"List all growth control genes in dogs" *is as follows.

select *GeneID*

from *Gene G, Protein P*

where *G.UniProtID = P.ProteinID *and *G.Organism = "Dog" *and *P.Function = "growth control"*

Given the database instance Δ, this query will produce the response *LOC608882*. In the event the third row in table *Gene *is missing, our hope is that we still will be able to compute this response because we know (from the rules in  I) *Q9NVA1 *is a *growth control *protein encoded by gene *UQCC *which is an ortholog of *LOC608882*. Since we would not know to which organism *LOC608882 *belongs, we could invoke a function, say *OrganismMapper*, that maps a gene ID to an organism, and see if *LOC608882 *belonged to *dog*. If such a function existed in the database, the query generator would also return the following SQL query segment that we would union with the one above to generate all possible responses.

select *GeneID*

from *Gene G, Protein P, EquivelantGenes E*

where *G.UniProtID = P.ProteinID *and *G.GeneID = E.Object1 *and *P.Function = "growth control" *and *OrganismMapper(E.Object2) = "Dog"*

In [30], we introduced an SQL construct using which external web form based, or desktop based, functions may be conveniently used as tables to maintain compatibility with SQL. This mechanism allows the capability to exploit arbitrary external resources to enhance computing capabilities. For example, consider now the query *"List all growth control genes in dogs that function similar to human genes"*. This query is more complex and requires more analysis because a direct conclusion is not possible from the database instance. A possible approach is as follows. First, find the growth control gene *LOC608882 *in dog. Then, find all the orthologs of this gene in human. Check to see if they control growth. Else, find all genes in human and find DNA similarity, and infer functional similarity using tools. Return response found. Clearly, the complexity has increased, but if the knowledge to carry out the process is included in the ontology, constructing the required query is possible.

## Results and discussion

**Figure 6:**
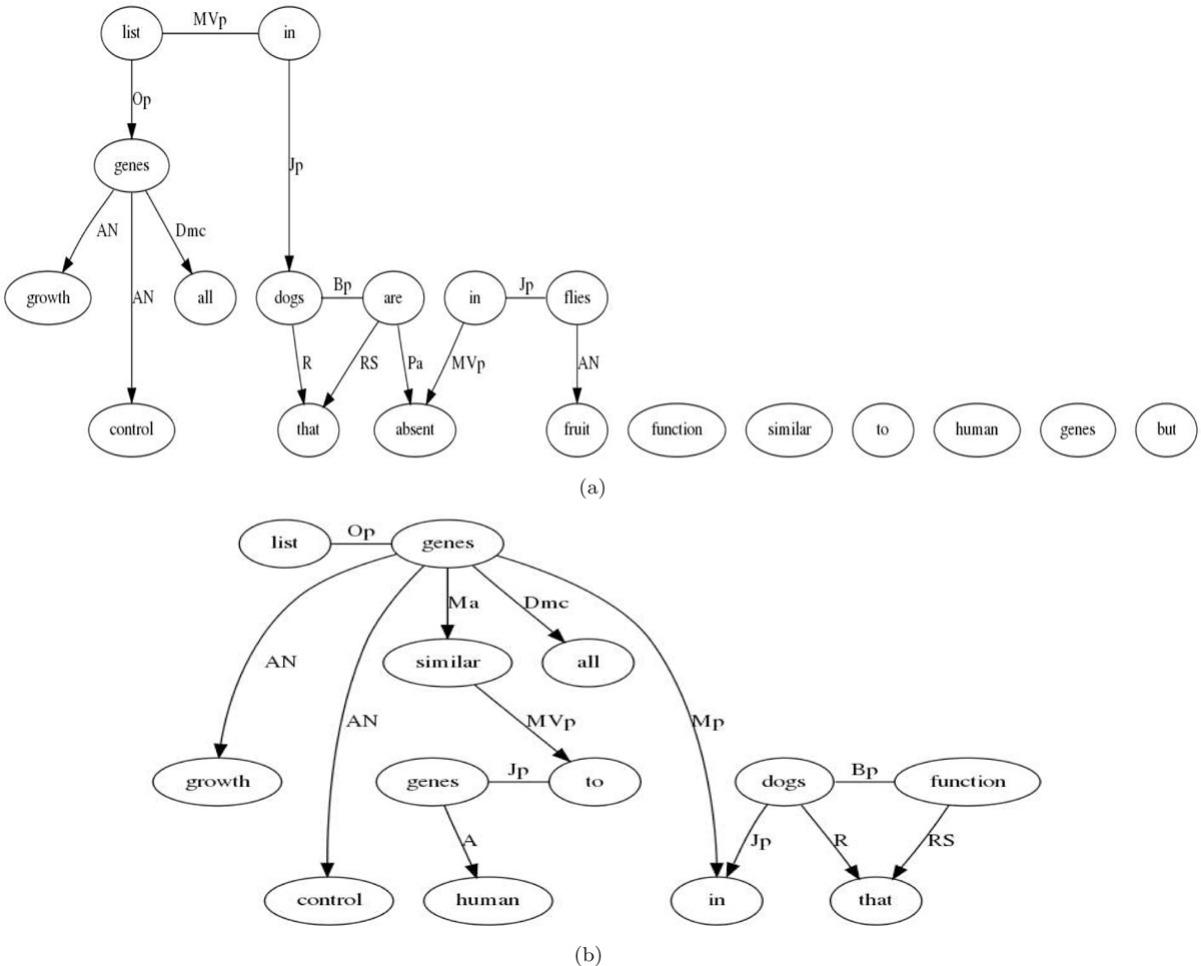
**Query parse trees**.

At this point, it is only instructive to mention that the entire query *"List all growth control genes in dogs that function similar to human genes but are absent in fruit files" *though feasible, is likely to be very complex if effective semantic role of the sentence can be computed. Our attempt to compute a dependence graph for this sentence using CMU Link Parser [42] is shown in Figure 6. CMU Link Parser has similar functionality as Stanford parser and MINIPAR but generates a visualization of the parse graph. The graph for this query is shown in Figure 6(a). It is apparent that for complex queries such as this, the dependence graph generation is a challenge. The figure shows broken links for many of the query terms. For less complex queries such as *"List all growth control genes in dogs that function similar to human genes"*, the parsers work reasonably well as shown in Figure 6(b). So the choice of the parsing and semantic link generation tool becomes exceedingly crucial. However, this choice often forces changes in our system as well as significant interfacing effort. In our current prototype, we remain uncommitted to a specific parser as we investigate several parsers and their suitability for such complex queries.

We believe the novelty of our plug-in is in its ability to bridge the semantic gap between a database schema and engine that maps NLP queries into queries in SQL or other declarative languages by leveraging the parsing effectiveness of short NLP queries by Stanford Parser or MINIPAR. To accurately map each NLP query to an equivalent SQL query, we leverage a high level conceptual description of an application in the context of which all NLP queries are interpreted. The dynamic nature of the plug-in allows updates in the knowledge base to refine application semantics to improve the quality of the query response. Such flexibility is desirable in databases needing to change the underlying database schema, or the guiding rules for query response. The separation of layers in our system also allow for accommodating changes at any level without disturbing the other layers, thus facilitating the portability of our approach to other applications.

## Conclusions

Our goal in this paper was to explore the outline of a smarter and cooperative natural language interface for biological database where the semantics of a query usually have many interpretations. We have demonstrated that in such databases, even though an answer is available, traditional systems will usually fail to respond due to the conventional query processing model. The novelty of our approach is in the generation procedure of the schema graph that embodies the semantic relationship in a conceptual ontology in terms of an arbitrary database scheme in a user transparent way. The process ensures that all the information content of a database is matched to the conceptual view to the maximum possible extent. The inclusion of conceptual rules enhances the querying capability of the system and allows deductive queries involving complex rules and external functions, even over-the-internet queries. This feature now allows users to plug in generic hypotheses and enrich the query capability of the engine, modifying as needed. Our contention is that the idea of an automatically generated schema graph allows us to map natural language queries to SQL queries over arbitrary database scheme. The requirement that we include an ontology and a type hierarchy is far less restrictive and non-technical than the requirements imposed by contemporary systems (concrete schema correspondence, application specific dictionary, restriction on the type and structure of English queries, etc.). These two requirements can be readily met by many domain ontologies being proposed and developed by the community, one of which is Gene Ontology. From the standpoint of community knowledge sharing, this is a significant strength of our system.

This paper also raises an interesting question - is it possible to transform an SQL query to respond in a way similar to how it would respond to a natural language query. For example, is it possible to transform the SQL query discussed earlier that one would traditionally issue to compute the query *"List all growth control genes in dogs"*. We assert that it is easier to provide a smarter response when a natural language query is asked because such queries offer the semantic richness to explore logical alternatives, that is seemingly difficult at the SQL level. However, this is one of the issues we would like to explore in the future.

## Competing interests

The author declares that they have no competing interests.

## Authors' contributions

HMJ is the sole contributor of this paper.
